# A preliminary study on the relationship between symptom severity and age of diagnosis in females versus males with autistic spectrum disorder

**DOI:** 10.3389/fpsyg.2025.1472646

**Published:** 2025-02-10

**Authors:** Alberto Sánchez-Pedroche, Eva Aguilar-Mediavilla, Mario Valera-Pozo, Daniel Adrover-Roig, Magdalena Valverde-Gómez

**Affiliations:** ^1^Department of Applied Pedagogy and Educational Psychology, Institute for Educational Research and Innovation (IRIE), University of the Balearic Islands, Palma, Spain; ^2^Institute of Child and Adolescent Mental Health, Can Misses Hospital, Ibiza, Spain

**Keywords:** ASD, female gender, diagnosis, severity index, child and adolescent, sex differences

## Abstract

In the latest autism observation, autism spectrum disorder (ASD) is more frequently diagnosed in males than in females. Efforts have been made in recent years to detect specific clinical patterns in females, improving their detection and diagnosis. Despite advancements, there are still challenges in detecting ASD in females. This preliminary study explores whether the age at the time of diagnosis of ASD in females is related to a higher severity index compared to male participants. A total of 202 participants (52 females; *M* = 5.51) in Spain underwent ADOS-2 assessment for ASD clinical severity. The results indicate a significant inverse association between the severity index and the age of diagnosis, which was independent of sex. Despite this, the present results revealed that the age at diagnosis was higher in females compared to males. Further analyses also revealed a tendency toward higher severity levels in females. Factors contributing to this difference in the age of diagnosis of ASD between females and males are discussed concerning the presumed differential characteristics of ASD in females.

## 1 Introduction

Autism spectrum disorder (ASD) is a neurodevelopmental condition characterized by persistent difficulties in social communication and restrictive repetitive patterns of behavior, interests, or activities. According to the Diagnostic and Statistical Manual of Mental Disorders, Fifth Edition (American Psychiatric Association [APA], 2014), ASD includes a range of symptoms that vary in intensity and presentation in each individual. The symptoms are present early in development, but they may not fully manifest until social demands exceed the child’s limited abilities or are masked by learning strategies (World Health Organization, 2019). In the DSM-5, the severity is based on impairments in social communication and restricted and repetitive patterns of behavior, ranging from grade 1 (requiring support) to grade 3 (requiring very substantial support). This variability has led to the term “spectrum” in the grading of this disorder.

The prevalence of ASD varies in different studies and populations. Based on the most current data available, the prevalence data is 1 in 36–2.78%- (CDC, Maenner et al., 2023), similarly to a recent systematic review of the global prevalence of ASD, which reports a range of 0.1 to 2.9% (Zeidan et al., 2022). Nevison and Zahorodny (2019) further analyzed trends in ASD prevalence across different racial and ethnic groups, showing significant variations in diagnostic rates over time. Regarding Spain, the estimated prevalence of diagnosed ASD among preschool children is 2.52%, while the prevalence among primary school children was lower (1.12%), and the ratio between boys and girls was 4:1 (Morales-Hidalgo et al., 2018). Among girls, the prevalence ranged from 0.39 to 0.58%, which is much lower than the overall 1% prevalence of ASD. This difference may reflect that diagnostic efforts and tools are more focused on early childhood, leading to higher detection rates in preschool children. Additionally, some children, particularly females, may camouflage symptoms or present subtler characteristics, delaying diagnosis until adolescence or adulthood (Hull et al., 2017; Rutherford et al., 2016).

Preschool-aged children in Spain are diagnosed at around 45 months, and elementary school children at around 87 months (Hernández et al., 2005). These results reflect a high number of diagnoses in the early stages of childhood, indicating a tendency toward an early diagnosis of ASD in Spain, which may unveil more severe patterns in preschool-aged children. A disparity has been observed in the prevalence of ASD depending on sex, being diagnosed more frequently in boys as compared to girls (Van Wijngaarden-Cremers et al., 2014). However, this difference in sex-related prevalence has been the subject of debate since girls and women with ASD often present different characteristics and manifestations that could go unnoticed under traditional diagnostic criteria (Werling and Geschwind, 2013). In the same vein, it is becoming increasingly evident that ASD may present differently in males compared to females (Wood-Downie et al., 2021). For a considerable time, the clinical focus and most of the research has been on the behavior manifestations typically observed in males with ASD, due to the higher prevalence of ASD in males. However, this research has the potential to introduce a bias in how the diagnosis of ASD is understood and manifested clinically and symptomatically in females (Montagut et al., 2018; Tofani et al., 2023). For example, girls with ASD may exhibit more nuanced social communication difficulties, such as an intense interest in people rather than objects, which may be misinterpreted as typical social behavior (Lai et al., 2015). Furthermore, they may engage in imaginative play, albeit with repetitive and scripted patterns, which may result in the symptoms being overlooked (Hiller et al., 2014). Furthermore, girls are more likely to disguise or conceal their symptoms, which may result in a higher rate of undetected cases (Hull et al., 2017). It is also important to note that females are more likely to receive a diagnosis later in adolescence or adulthood, particularly when symptoms are masked (Rutherford et al., 2016; Duvekot et al., 2017). In recent years, specific screening tools focusing on females with ASD, such as the ASSQ Girl (Kopp and Gillberg, 2011), have been developed to improve early detection and diagnosis in females. Despite these advances, challenges remain in the detection of ASD in girls, which may result in later diagnoses for those with lower severity rates.

Following the above revised literature, the main objective of this preliminary study was to analyze the relationship between the severity index and the age at the time of diagnosis. We expected that (1) girls would show a higher severity index and a higher age at diagnosis of ASD as compared to boys; (2) a larger severity index would be associated with an earlier age of diagnosis, particularly in girls.

## 2 Method

### 2.1 Participants and diagnostic protocol

We departed from an initial sample of 647 participants referred for diagnosis as possible ASD in the Balearic Islands (Ibiza and Formentera, Spain) during the years 2020–2024. All participants were assessed in accordance with the comprehensive clinical protocol of the Balearic Institute of Child and Adolescent Mental Health (IBSMIA), that includes the consideration of each participant’s developmental history and the screening with the Modified Checklist for Autism in Toddlers (M-CHAT; Robins et al., 2009), the Social Communication Questionnaire (SCQ; Rutter et al., 2003) and the NICE (National Institute for Health and Care Excellence) tests, and subsequently administered the gold standard ADOS-2 test (Lord et al., 2015). Four hundred and forty-five participants did not meet the clinical criteria for a diagnosis of ASD and were discarded from the final sample. A total of 202 participants (52 females) with a mean age of 5.49 years (*SD* = 4.063, range 1–16 years) were included in the final sample. All participants older than 3 years were enrolled in mainstream schools.

A power analysis indicated that a sample size of approximately 103 participants per group is required to detect a small to medium effect size (*d* = 0.39) with a significance level of 0.05 and a statistical power of 0.80. As expected, due to the inherent imbalance in autism prevalence between males and females, the final sample included 150 males and 52 females. Although the female group did not meet the benchmark set by the power analysis, this sample size remains substantial in the context of autism research, where females are significantly underrepresented. Furthermore, the total sample size (*N* = 202) is close to the combined requirement of 206 participants, supporting the validity of the statistical analyses performed.

### 2.2 Materials

To assess the severity of ASD symptoms, we used the Autism Diagnostic Observation Schedule, ADOS-2 (Lord et al., 2015). This scale is one of the most used instruments for ASD diagnosis in clinical practice, becoming a gold standard. The ADOS-2 is a standardized semi-structured direct observation scale designed to detect social and communicative behaviors within the autism spectrum, as well as characteristics of restricted repetitive and stereotyped behaviors. The ADOS-2 samples quantify individual’s behaviors and provides a clear diagnostic score as well as severity scores. It consists of five different modules depending on the level of expressive language and/or the age of the individual, and several algorithms that combine specific scores in several items observations to classify the assessed behaviors. Each algorithm has specific criteria that are applied during the evaluation and are used to assign a final score or categorization on the ASD. The algorithm totals are compared to the diagnostic classification limits. The ADOS-2 is one of the best psychometrically (specificity 95%, sensitivity 95%, inter-rater reliability 84%) evaluation tools in the diagnosis of ASD and has excellent diagnostic validity according to multiple studies (Zander et al., 2016).

### 2.3 Procedure

This study was conducted in accordance with the ethical guidelines set forth by the relevant authorities, and all data pertaining to the participants were collected as part of the routine clinical evaluations. The participants were recruited from the IBSMIA (Balearic Institute of Mental Health for Children and Adolescents), a public service where children are assessed to determine the presence of ASD or other developmental difficulties. Prior to their participation, written informed consent was obtained from the parents or legal guardians, which included a clause authorizing the use of anonymized data for research purposes.

All assessments were conducted in four 60-min sessions at the IBSMIA facilities. Two researchers from the research team possess official clinical accreditation for the administration of the ADOS-2, having obtained it through IGAIN (University of Barcelona) and the “Hospital Universitari Mútua de Terrassa.” To guarantee the fidelity of administration, the research team held regular peer review sessions to supervise adherence to standardized procedures and scoring consistency.

For the ADOS-2, the raw data were transformed into a Calibrated Severity Scores ranging from 0 to10 following the mapping table of the ADOS-2 as compiled by Gotham et al. (2009). This mapping table standardizes raw ADOS totals into severity scores for consistent interpretation (see Table 1). This classification framework was employed exclusively for research purposes, with the objective of facilitating comparisons between groups. It does not supersede the clinical judgment necessitated for DSM-5 diagnostic criteria, which are predicated on the level of support required by the individual (American Psychiatric Association [APA], 2014). This table also allows the classification of the Calibrated Severity Score into Severity Levels. These levels correspond to the diagnostic classification of autism (American Psychiatric Association [APA], 2014), being the following: calibrated severity scores ranging from 1 to 3 (both included) correspond to a non-diagnosis of ASD and were not considered for the final sample; calibrated severity scores between 4 and 5 would correspond to the level 1 ASD diagnostic category and was coded as 2; calibrated severity scores between 6 and 8 would correspond to the level 2 ASD diagnostic category and was coded as level 3; and finally, calibrated severity scores between 9 and 10 would correspond to the level 3 ASD diagnostic category, and was coded as 4.

**Table 1:** Sex-dependent distribution of severity level.

Severity level	*N* male (%)	*N* female (%)
2	21 (14%)	8 (15.4%)
3	111 (74%)	31 (59.6%)
4	18 (12%)	13 (25%)

Severity levels derive from the calibrated severity scores. Calibrated severity scores between 4 and 5 correspond to ASD level 1 and were coded as 2; calibrated severity scores between 6 and 8 correspond to ASD level 2 and were coded as 3; and calibrated severity scores between 9 and 10 correspond to ASD level 3 and were coded as 4.

The SPSSv.28 statistical package was used for data analysis. As the Calibrated Severity Score is a categorization of the raw data of ADOS-2, we used a Spearman Rho correlation to assess the relation between this variable and the age at the diagnostic in all sample and split by sex. We also performed two *t*-tests to compare the Calibrated severity Score and the age of diagnostic between males and females; and *χ*^2^ to compare the frequency distribution of the severity index between males and females. Finally, we used an ANOVA (2 × 3) with the factors Sex and Severity Level on the age of diagnostic.

## 3 Results

Table 2 shows the descriptive statistics for the main variables of the present study split by sex.

**Table 2:** Descriptive statistics.

	N	Age at diagnostic		Calibrated severity score	
		*M*	*SD*	*M*	*SD*
Female	52	6.65	4.85	7.21	1.64
Male	150	5.08	3.68	7.01	1.40
Total	202	5.49	4.06	7.06	1.46

The mean age at diagnosis for males was lower than for females [*t*(200) = −2.436, *p* = 0.016, *d* = 0.39]. Contrary, the Calibrated Severity Score mean did not show significant differences between males and females [*t*(200) = −0.839, *p* = 0.402, *d* = 0.14].

The results show a significant negative relation between the age at the diagnosis and the Calibrated Severity Score according to the ADOS-2 for the whole sample (*rho* = −0.168, *p* = 0.017), indicating that a higher severity score is associated with a younger age of diagnosis. Although the correlation value was slightly higher for females (rho = −0.207, *p* = 0.141), only a significant negative correlation was found in males (*rho* = −0.173, *p* = 0.019), probably due to the lack of statistical power given the moderately low number of females in the final sample. Accordingly, Fisher’s *Z* conducted to examine whether the correlation pattern differed between males and females was non-significant (*Z* = −0.21; *p* = 0.831). Thus, associations between the Calibrated Severity Score and the age of diagnosis were independent of sex.

To further explore the distribution of Severity Levels and Sex (Table 1), contingency analyses were conducted, and revealed a tendency of higher Severity Levels in females relative to males [*χ*^2^(2) = 5.44, *p* = 0.066].

**Figure 1:**
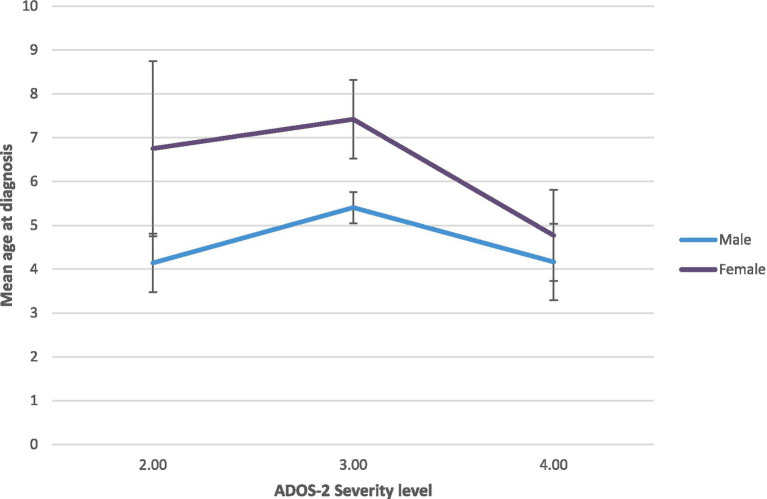
Age of diagnostic of ASD depending on calibrated severity score and sex.

Complementarily, a two-way ANOVA was conducted to examine the effect of Sex and Severity Level on Age at Diagnosis (see Figure 1). The results showed a significant main effect of Sex on Age at the Diagnosis [*F*(1,201) = 4.96, *p* = 0.027, *η*^2^ = 0.025], but only a tendency toward significance in the Severity Level [*F*(2,201) = 2.897, *p* = 0.058, *η*^2^ = 0.029], and a non-significant interaction between Sex and Severity Level [*F*(5,201) = 0.494, *p* = 0.611, *η*^2^ = 0.005].

## 4 Discussion

According to the present study, our results support a difference in the age of diagnosis between females and males, being females diagnosed later as compared to males. Nevertheless, there is only a tendency for females to be more frequently classified into higher Severity Level categories of ASD compared to males, as measured by the ADOS-2. Besides, the relationship between symptom severity and age of diagnosis appears to be independent of sex, as reflected by the lack of interaction between these variables (see discussion about hypothesis 2 below). Despite the lack of interaction, a later age of diagnosis and a higher tendency in females to fall into the most severe categories according to the ADOS-2 may suggest a potential sex bias in diagnostic practices (Gesi et al., 2021). Therefore, these results aligned partially with several previous studies (Constantino and Charman, 2012; Giarelli et al., 2010; Van Wijngaarden-Cremers et al., 2014) that have also shown a sex bias in ASD diagnosis. These findings underscore the necessity of elucidating the sex-specific clinical manifestations. Late diagnoses in females may be associated with less overt, intricate symptoms or the capacity to disguise social challenges, as evidenced in prior investigations (Hull et al., 2017; Lai et al., 2015; Tofani et al., 2023). These observations highlight the need for sex-sensitive diagnostic approaches to improve early detection in females. Therefore, our first hypothesis, which stated that girls would show a higher severity index and be diagnosed with ASD at an older age than boys, was partially supported.

Besides, the present results reflect that there is a significant negative correlation between severity and age of diagnosis, although this correlation was not dependent on sex. This also partially supports our second hypothesis that a larger severity index would be associated with an earlier age of diagnosis, although undifferentiated between females and males. This was also confirmed by the lack of interaction between sex and severity over the age of diagnosis. These results differ somewhat from previous findings (Constantino and Charman, 2012; Giarelli et al., 2010; Van Wijngaarden-Cremers et al., 2014) which suggest that females require a greater degree of impairment than males to receive a diagnosis. The present results suggest a sex-biased diagnosis in females that is relatively independent of the severity level. This aligns with several previous findings showing that females are less likely to be correctly diagnosed with ASD and more likely to be misdiagnosed at first evaluation than men (Gesi et al., 2021).

Factors contributing to the higher age to be diagnosed with ASD in females relative to males may include a different manifestation of ASD in females than in males with less typical, atypical, and visible symptomatology than in males. For example, Van Wijngaarden-Cremers et al. (2014) reported significant sex differences with higher levels of repetitive and stereotyped behavior in males than in females. This lack of visibility could delay the diagnosis till females grow and new manifestations are added (Gesi et al., 2021; Mandy et al., 2012; Rutherford et al., 2016). Further discrepancies may be explained by additional factors, including societal biases in recognizing ASD symptoms in females or differences in developmental trajectories (Brickhill et al., 2023). It would be beneficial for future studies to consider broader cultural and socioeconomic influences on diagnostic timing (Begeer et al., 2013; Dworzynski et al., 2012).

Despite this sex bias in the age of diagnosis, probably a potentially real sex difference in ASD prevalence (Werling and Geschwind, 2013), as well an increased awareness in recent years about the detection of ASD in females and their distinct presentation profiles have also influenced the present results. Consequently, an increase in knowledge about the differential manifestation of ASD in men and women is needed to identify specific patterns of clinical presentation in females and improve both detection and diagnosis in females (Wood-Downie et al., 2021; Van Wijngaarden-Cremers et al., 2014).

Our results show some limitations. Although the sample size is sufficient based on the power analysis, a larger sample would help to draw more robust and generalizable conclusions about the prevalence and age of diagnosis of ASD in female population. A further limitation pertains to the unequal sample sizes between males and females, which mirrors the inherent sex-based discrepancy in autism prevalence. Despite the female group exhibiting smaller sample sizes than the benchmark stipulated in the power analysis, it remains a substantial and meaningful sample within the context of autism research. This is particularly salient when one considers the challenges associated with the recruitment of females with autism. This disparity has been acknowledged, and it is emphasized that the findings should be interpreted with caution, particularly in the context of generalizing sex-specific differences.

Besides, including additional factors such as the socioeconomic status of the family and parental concerns in future analyses could provide a more comprehensive understanding of the variables influencing the age at diagnosis. We acknowledge that the current study did not include these factors, which may limit the generalizability of the findings, and for this reason it may have to be taken with caution. Longitudinal studies would also be beneficial to track changes in diagnostic criteria and their impact on sex differences in ASD diagnosis over time.

A further limitation of the study is its exclusive reliance on ADOS-2 scores for the assessment of symptoms severity. This decision was taken to ensure objectivity and consistency in comparing severity of symptoms between male and female participants. However, it must be noted that cut-off scores for ADOS-2 are primarily based on male participants, which might reflect the current disproportion in the prevalence of ASD between males and females. This limitation must also be considered when interpreting findings, particularly the comparison of symptom severity levels across sexes. Furthermore, the use of specific screening tools focused on females with ASD, such as ASSQ Girl (Kopp and Gillberg, 2011), could help improve early detection and diagnosis in females.

In conclusion, our study supports the existence of a sex difference in the age of ASD diagnosis and a tendency toward a distribution of higher severity levels in girls than in boys. The present findings highlight the need for continued research and awareness of the unique presentation of ASD in females.

## Data Availability

The raw data supporting the conclusions of this article will be made available by the authors, without undue reservation.
